# Active Terahertz Modulator and Slow Light Metamaterial Devices with Hybrid Graphene–Superconductor Photonic Integrated Circuits

**DOI:** 10.3390/nano11112999

**Published:** 2021-11-08

**Authors:** Samane Kalhor, Stephen J. Kindness, Robert Wallis, Harvey E. Beere, Majid Ghanaatshoar, Riccardo Degl’Innocenti, Michael J. Kelly, Stephan Hofmann, Hannah J. Joyce, David A. Ritchie, Kaveh Delfanazari

**Affiliations:** 1 James Watt School of Engineering, University of Glasgow, Glasgow G12 8QQ, UK; 2658952k@student.gla.ac.uk; 2 Cavendish Laboratory, University of Cambridge, Cambridge CB3 0HE, UK; sjk80@cam.ac.uk (S.J.K.); rw497@cam.ac.uk (R.W.); heb1000@hermes.cam.ac.uk (H.E.B.); mjk1@cam.ac.uk (M.J.K.); dar11@cam.ac.uk (D.A.R.); 3 Laser and Plasma Research Institute, Shahid Beheshti University, Tehran 19839 69411, Iran; m-ghanaat@sbu.ac.ir; 4 Department of Engineering, University of Lancaster Bailrigg, Lancaster LA1 4YW, UK; r.deglinnocenti@lancaster.ac.uk; 5 Engineering Department, University of Cambridge, Cambridge CB3 0FA, UK; sh315@cam.ac.uk (S.H.); hannah.joyce@eng.cam.ac.uk (H.J.J.)

**Keywords:** hybrid photonic integrated circuits, graphene, superconductors, terahertz photonics, terahertz electronics, electromagnetic induced transparency, slow light devices

## Abstract

Metamaterial photonic integrated circuits with arrays of hybrid graphene–superconductor coupled split-ring resonators (SRR) capable of modulating and slowing down terahertz (THz) light are introduced and proposed. The hybrid device’s optical responses, such as electromagnetic-induced transparency (EIT) and group delay, can be modulated in several ways. First, it is modulated electrically by changing the conductivity and carrier concentrations in graphene. Alternatively, the optical response can be modified by acting on the device temperature sensitivity by switching Nb from a lossy normal phase to a low-loss quantum mechanical phase below the transition temperature (*T*_c_) of Nb. Maximum modulation depths of 57.3% and 97.61% are achieved for EIT and group delay at the THz transmission window, respectively. A comparison is carried out between the Nb-graphene-Nb coupled SRR-based devices with those of Au-graphene-Au SRRs, and significant enhancements of the THz transmission, group delay, and EIT responses are observed when Nb is in the quantum mechanical phase. Such hybrid devices with their reasonably large and tunable slow light bandwidth pave the way for the realization of active optoelectronic modulators, filters, phase shifters, and slow light devices for applications in chip-scale future communication and computation systems.

## 1. Introduction

Metallic superconductors are macroscopic quantum systems and gain their electromagnetic properties from pairs of electrons, Cooper pairs [1]. Due to their intrinsic low-loss and plasmonic properties, they are excellent platforms for applications, especially in cryogenic nano-electronics and nano-photonics [2,3,4,5,6]. Graphene is a thin layer of carbon atoms arranged in a hexagonal network. It is a two-dimensional (2D) material, the thinnest example of a material [7]. The combination of 2D materials and superconductors offers novel electronic and photonic properties that may not be found in either of these materials independently [8,9]. For example, it is possible to measure the superconducting gap in graphene when it is placed in close and clean proximity to a host superconducting material, such as niobium (Nb), via the proximity effect [10]. A proximitized graphene then may show exotic electronic or photonic properties, such as topological phases, that are robust against weak external perturbation and are proposed for applications in fault-tolerant quantum computing [11].

The surfaces of hybrid materials can be engineered to achieve electromagnetic response at will. Such engineered materials, metamaterials, develop their exotic electromagnetic properties from the geometries of the engineered unit cells (artificial atoms) through the interactions of artificial atoms and photons [12]. For example, electromagnetically induced transparency (EIT) has been found to have a classical analogy using metamaterials [13,14]. EIT is a nonlinear quantum effect that evolves due to the destructive interference between excitation states in three-level atomic systems, which results in a narrow transparency window in the medium where light can pass through without any absorption [15]. The classical analogue of EIT observable in integrated metamaterial devices offers an extreme modification of dispersion properties resulting in group delay enhancement, slow down, and storage of light [13,16].

An integrated optoelectronic device with the capability of continuous tuning and controlling of the group velocity of light is therefore of interest in the microwave, millimeter wave, terahertz (THz), and far infrared regime, which make possible, for example, the realization of (i) tunable optical phase shifter, (ii) time-delay lines [13] as tools to control the emission of the optoelectronic and telecommunication systems, and (iii) continuously tunable fast bandpass filter or bandstop filter [17]. Such devices are also very useful tools for actively controlling the dispersion as external cavity mirrors for ultrashort pulsed QCLs [18,19,20]. Moreover, they can be used for active modulation and polarization control of superconducting THz quantum emitters [21,22,23,24,25,26,27,28,29,30,31,32,33]. The demonstration of ‘*static*’ superconducting THz metamaterials was reported where the authors observe tuning response with intense THz light [34,35], temperature [13,31,36,37,38,39], and applied DC bias voltage [40]. Metamaterials with asymmetric couplings within a pair of static gold and normal metal split ring resonators (SRRs) have also been studied to control the THz wave [41,42].

In this work, a single layer of graphene is integrated with superconducting Nb plasmonic SRRs to realize a voltage and temperature tunable ‘*active*’ slow light optoelectronic device operating at cryogenic temperatures. The advantages of our proposed devices include: (i) Nb as a metallic superconductor obtains quantum mechanics phases at cryogenic temperatures to offer active modulation and tuning of THz response in wide temperature and frequency ranges. Such sensitive temperature sensing, as a result of the temperature-dependent superfluid density of the Nb superconductor, cannot be achieved with any normal or noble metal-based metamaterial devices. (ii) The current most promising platform for a quantum computer is based on superconducting processors operating at cryogenic temperatures. Therefore, it is necessary to develop tunable slow light devices, sensors, detectors, and modulators operating at low temperatures. (iii) The combination of graphene as a 2D material with their tunable carrier concentration with voltage offers an additional knob to control light in such novel integrated circuits at cryogenic temperatures.

## 2. Design of the Hybrid Photonic Integrated Circuits

**Figure 1:**
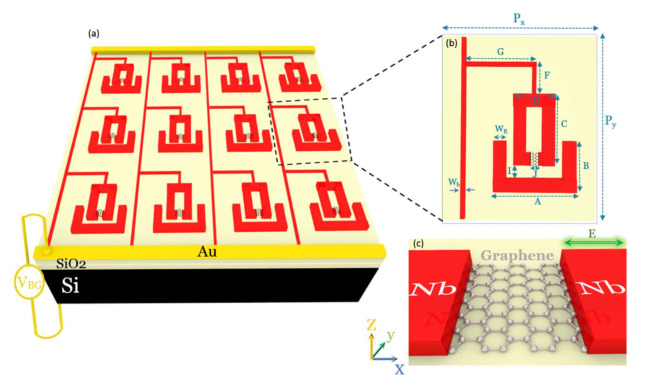
Voltage-controlled hybrid graphene–superconductor THz photonic integrated circuit. (**a**) The hybrid device architecture shows arrays of coupled graphene-Nb SRR integrated with superconducting circuits. Here, Nb, Au, SiO_2_, Si, and graphene are shown in red, yellow, gold, black, and gray, respectively. (**b**) A meta-atom (unit-cell of the device). (**c**) The magnified gap structure of a meta-atom, showing graphene is sandwiched between two Nb fingers in the upper resonator in the superconducting circuit. Polarization of the incident THz wave is shown by a green arrow.

The architecture of the proposed hybrid graphene–superconductor photonic integrated circuit is schematically shown in Figure 1a. The device contains a two-dimensional array of coupled SRRs, oppositely facing each other, in a superconducting circuit. A single layer of graphene is integrated with the top resonator in its small finger (capacitor gap). The superconducting material is considered to be Nb. The THz slow light device with a tunable THz superconductor–graphene–superconductor resonator can be modulated (i) thermally by switching from a lossy metallic to a low-loss superconducting medium due to the increase in the Cooper-pairs densities below the transition temperature (*T*_c_ = 9.2 K) of Nb and (ii) electrically by changing the conductivity and carrier concentrations in graphene.

Figure 1b shows a meta-atom in the device with dimensions set to be: A = 52 μm, B = 42.4 μm, C = 46 μm, D = 26.8 μm, F = 15.6 μm, G = 39.6 μm, I = 8 μm, the upper SRR gap length of J = 4 μm (distance between two Nb fingers), the width of W_R_ = 8 μm, and bias line width of W_b_ = 4 μm. The unit cell periodicity is set to be (P_y_ = 114.4 µm) × (P_x_ = 76 µm). The single unit cell is repeated into arrays to ease light coupling. The thickness of Nb is considered to be 100 nm. Figure 1c depicts the magnified gap structure of the top resonator, showing that graphene is sandwiched between two Nb fingers of the top SRR. The polarization of the incident THz wave is shown with a green arrow in Figure 1c. The device is designed based on a 500 µm thick boron p-doped silicon Si substrate with a 300 nm insulating layer of SiO_2_. The THz transmission of bare SiO_2_/Si substrate is used as a reference. In order to design and model the device architecture, the RF module of COMSOL Multiphysics V5.5 is used. SiO_2_ and Si are considered dielectrics with *ε* = 3.9 and 11.56, respectively [43]. In the low THz frequencies (this work), only the intraband contribution of absorption is considered in the graphene conductivity [44]. At DC, relaxation effects of free charges are described via the DC conductivity $\sigma_{0}$ [45]. The AC conductivity is related to the DC conductivity through the Drude model $\sigma_{G}\left(\omega \right)=\frac{\sigma_{0}}{1+i\omega \tau }$, where *τ* = 15 *f*s is the scattering time, and *ω* is the angular frequency [44,46]. The intraband part was found to remain almost temperature-independent [47,48]. The DC conductivity of graphene $\sigma_{0}$ is set to be between 0.3 mS and 2.5 mS in the simulation. For describing the variation of graphene conductivity, only the DC conductivity *σ*_0_ is mentioned in the paper. Graphene is simulated as a 2D layer with surface current boundary conditions. The surface current of graphene $J=\sigma_{G}E$ is defined as the product of its conductivity ($\sigma_{G}$) in Siemens unit and the tangential electric field (E). The experimental complex conductivity of Nb is described in the framework of Mattis–Bardeen equations with a finite scattering rate. The imaginary part of conductivity of Nb is much higher than the real part, which gives rise to the superconductor’s dominance reactive response [13,49,50]. The imaginary part shows a 1ω dependence and rises when entering the superconducting phase. The dissipative real part of conductivity shows a minimum representing the energy gap of the superconducting state in the frequency spectra, which opens up at the transition temperature, shifts to a higher frequency as the temperature decreases, and reaches its maximum at 0.7 THz for 4.5 K. On the other hand, the conductivity of a normal metal is in accordance with the Drude model. With respect to the model, the imaginary part of conductivity is much smaller than the real part, which results in a near-zero value of kinetic inductance of normal metals [49]. Along with it, the frequency dependency of conductivity in a normal metal is lower than in a superconductor [50]. The conductivity of Au ($\sigma_{\mathrm{Au}}$) is described by the Drude model expression as $\sigma_{\mathrm{Au}}=\varepsilon_{0}\frac{\omega_{p}^{2}}{\gamma +i\omega }$, where the plasma frequency $\omega_{p}$ is 2π×2175 THz, and the collision frequency γ is 2π×6.5 THz [51]. Here, $\varepsilon_{0}$ is the vacuum electric constant.

## 3. Results and Discussion

### 3.1. Superconducting THz Photonic Circuits without Graphene Patches

**Figure 2:**
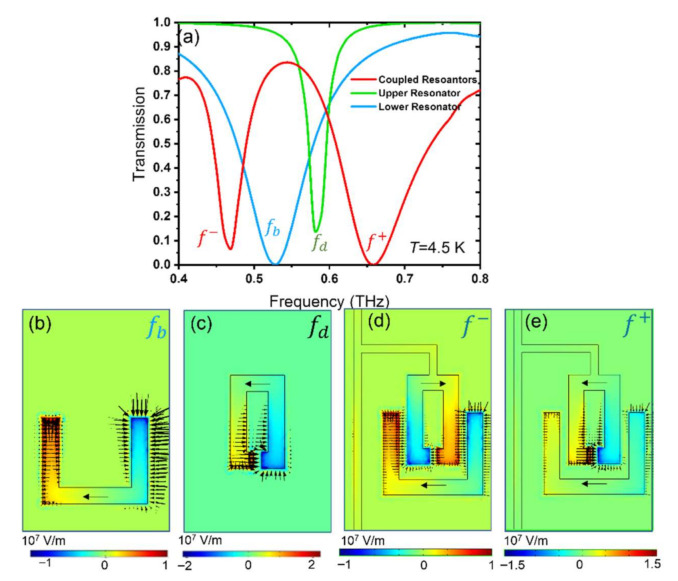
(**a**) Simulated THz transmission of static superconducting Nb SRR arrays in the absence of graphene. Transmission curves when only a bottom ring structure (blue), when only a top ring structure (green), and when two coupled ring resonators (red) are used at *T*= 4.5 K. (**b**–**e**) The *z*-component of the electric field *E*_z_ and surface current distribution at relevant resonance frequencies, shown in (**a**).

We first consider the THz response of three sets of static superconducting SRRs in the absence of graphene, at *T* = 4.5 K, far below the transition temperature *T*_c_ of Nb. The THz transmissions of three sets of SRRs are shown in Figure 2a. The geometries of the three sets of SRRs are shown in Figure 2b, here called the bottom single resonator, Figure 2c, the top single resonator, and Figure 2d, as coupled resonators that are faced oppositely (rotated by 180 degrees with respect to each other). In the absence of coupling between two resonators, each SRR independently supports localized surface plasmon (LSP) resonances [17,46]. They show a typical inductive-capacitive (LC) resonance, with center frequencies at 0.53 THz (labeled *f*_b_) and 0.58 THz (labeled *f*_d_), respectively. The bottom resonator’s longer side is directly excited by the incoming THz *E*-field. Therefore, the excited net dipole moments in the longer arm result in a resonance with a broader full width at half maximum bandwidth (FWHM), as the radiation damping is SRR structure size-dependent. The total length of the bottom resonator is also slightly larger than the top resonator resulting in resonance at a lower frequency.

The electric field distribution in the coupled resonators shows the strength of the resonators to incident THz radiation polarized along the *x*-direction, see Figure 2b–e. The smaller SRR (top) acts as a subradiant (or dark) resonator with resonance frequency *f*_d_. On the other hand, the larger SRR (bottom) acts as a superradiant (or bright) resonator with resonance frequency *f*_b_. However, the destructive interference between two subradiant and superradiant resonances leads to two dips as hybridized modes of “bonding” and “anti-bonding” and one peak as an asymmetrically coupled mode [52], as shown by the red curve in Figure 2a. The electric field and current distribution at the bonding *f* ^−^ and anti-bonding *f* ^+^ resonance frequencies are shown in the lower panel of Figure 2. The induced current directions in two coupled SRR are opposite for *f* ^−^ bonding mode, while they are in the same direction for *f* ^+^ anti-bonding mode. The suppressed currents due to strong asymmetric coupling and destructive interference between a subradiant mode from the top SRR and a superradiant mode from the bottom SRR lead to induced electromagnetic transparency (EIT) in the coupled SRRs [53].

### 3.2. Integration of Graphene with Superconducting THz Photonic Circuits

**Figure 3:**
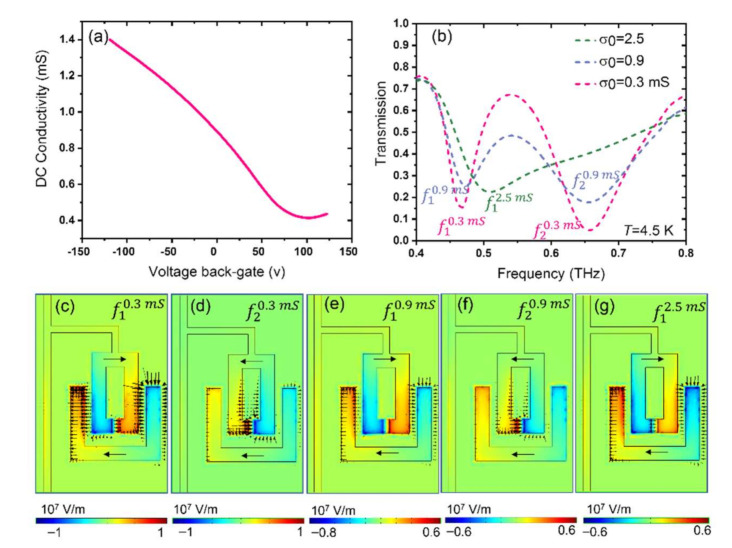
(**a**) The measured conductivity of graphene as a function of back-gate voltage; (**b**) Simulated THz transmission of the active/electrically tunable photonic integrated circuit with hybrid graphene–superconducting Nb SRR arrays for three different graphene conductivity, *σ*_0_ = 0.3 mS (dashed red), *σ*_0_ = 0.9 mS (dashed purple), and *σ*_0_ = 2.5 mS (dashed green). (**c**–**g**) The *z*-component of the electric field *E*_z_ and surface current distribution at the relevant resonance frequencies shown in (**b**).

Now, we discuss the results for the active, electrically tunable device based on the integration of graphene with Nb SRR arrays in a superconducting circuit. The preliminary graphene characterization of the continuous patch was carried out at room temperature and is shown in Figure 3a, providing us with range of conductivity that we assume for our metamaterial arrays as well. The graphene is initially p-doped; therefore, due to the excess of charge carrier already present in the graphene before the back-gate voltage, the Dirac point is at the voltage of 102 V for the given device [46,54].

The THz transmission spectra of the device with graphene in the finger of the top SRR, with lowest *σ*_0_ = 0.3 mS (close to Dirac point), *σ*_0_ = 0.9 mS (corresponding to gate voltage *V*_BG_ = 0 V), and highest *σ*_0_ = 2.5 mS conductivity values shown in dashed red, purple, and green in Figure 3b. By comparing Figure 2a and Figure 3b, one can see that the integration of graphene with dark resonator results in the increase in damping in the resonance of the dark resonator. The results suggest that by the electrostatic gating of graphene, one can actively tune the device resonance frequency between a strongly coupled-resonator circuit with super-radiant resonance *f*_1_^0.3mS^ and a single resonator LSP circuit with sub-radiant resonance *f*_1_^2.5mS^. Figure 2 and Figure 3 also show the blueshift of the *f* -mode by 41 GHz when the graphene conductivity changes from *σ*_0_ = 0 mS, a static resonator circuitry with no graphene in the dark resonator’s gap, to *σ*_0_ = 2.5 mS where the graphene conductivity is set to highest value (*f*_1_^2.5mS^ mode shown in dashed green). The electric field distribution of this mode shows the damping of the resonance at the dark element (see lower panel of Figure 3).

### 3.3. Modulation of THz Waves in Hybrid Graphene Superconducting Photonic Circuits with Temperature

**Figure 4:**
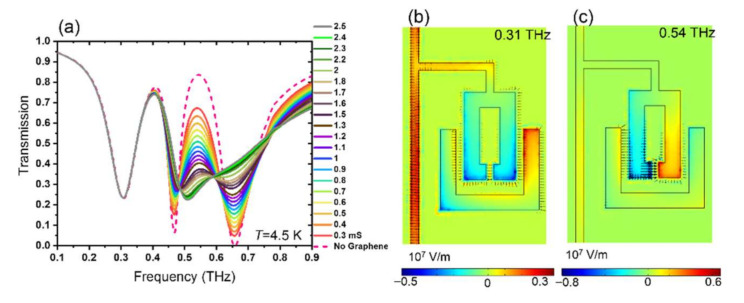
(**a**) Simulation transmission data for *T* = 4.5 K at different graphene DC conductivities. Z-component of electric field distribution at *σ*_0_ = 0 mS for (**b**) bias line resonance frequency *f* = 0.31 THz, and (**c**) the peak frequency *f* = 0.54 THz.

The device is designed in a way to have electric fields that are concentrated in the bright and dark resonators despite the introduction of the superconducting circuit (used to connect the dark resonators in the device and to bias the graphene patch). The bias line exhibits resonance at *f* = 0.31 THz for *T* = 4.5 K. The bias line resonance shows no variation with graphene DC conductivities (see Figure 4a). The electric field distribution at bias line resonance frequency in Figure 4b accumulates at the bias line. On the other hand, the electric field distribution at the peak mode in Figure 4c and also two dark and bright resonances (as shown in Figure 2 and Figure 3) show no enhancement at the bias line. Therefore, the bias line is designed to have a low influence on the EIT window between bright and dark resonances. The circuit exhibits a resonance at around 0.3 THz, so with less impact on the THz transmission response of the coupled resonator arrays.

**Figure 5:**
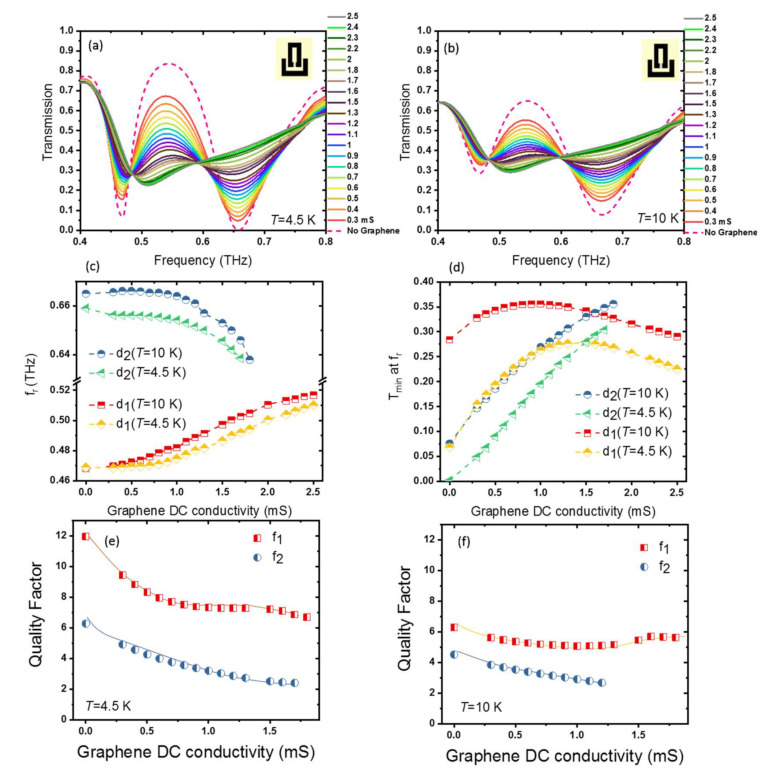
THz transmission of the metamaterial photonic integrated circuit with hybrid graphene-Nb SRRs for different graphene conductivities: (**a**) at *T* = 4.5 K, far from the Nb superconducting transition temperature *T*_c_, where Nb is in the superconducting quantum phase, and (**b**) at *T* = 10 K, where Nb is no longer in the superconducting quantum phase. (**c**) Resonance frequencies and (**d**) transmission dips at resonance frequencies of the metamaterial integrated circuit, as a function of graphene’s conductivity at *T* = 4.5 K and *T* = 10 K. (**e**) Quality factor at resonance frequencies at *T* = 4.5 K and (**f**) quality factor at resonance frequencies at *T* = 10 K.

To obtain further insight into the voltage-controlled active hybrid graphene–superconductor photonic integrated circuit demonstrated in this paper, we compare the detailed results for (i) when Nb is in superconducting quantum phase state below *T*_c_ and (ii) when Nb is in the resistive normal state above *T*_c_. The THz transmission spectra as a function of frequency for different graphene conductivities between *σ*_0_ = 0 mS and *σ*_0_ = 2.5 mS at *T* = 4.5 K < *T*c_Nb_ and at *T* = 10 K > *T*c_Nb_ are shown in Figure 5a,b, respectively. Figure 5c,d shows the resonance frequencies and transmission dips at resonance frequencies of the device, as a function of graphene conductivity at two different temperatures *T* = 4.5 K, and *T* = 10 K. A clear enhancement of THz transmission, superradiant and subradiant modes, and frequency tuning can be seen when the device is in superconducting quantum phase state at *T* = 4.5 K. Quality factor of first (*f*_1_) and second (*f*_2_) resonances for the temperatures *T* = 4.5 K and *T* = 10 K are shown in Figure 5e,f, respectively. With an increase in the graphene’s DC conductivity, the strength of both resonances decreases up to the *σ*_0_ = 1.8 mS, where the second resonance completely damps, and only one single resonance remains.

The resonances at *T* = 4.5 K are sharper, and hence, the device is more sensitive to incident THz light at the superconducting state than that at *T* = 10 K, where Nb obtains a normal metallic phase. The change of the transmission minimum at the first dip between graphene conductivity *σ*_0_ = 0 and *σ*_0_ = 2.5 mS for *T* = 4.5 K is 0.16, where it is 0.01 at *T* = 10 K. The variation for the second dip at *T* = 4.5 K between *σ*_0_ = 0 and *σ*_0_ = 1.7 mS is 0.3, which reduces to 0.27 for *T* = 10 K. The field concentration in the gap is more enhanced at *T* = 4.5 K. As a result of more interaction of electric field in the gap with graphene, the change in transmission at *T* = 4.5 K is larger than that at *T* = 10 K. This suggests that our hybrid superconducting THz devices are suitable for THz sensing at wide temperature ranges below the *T*_c_ of Nb.

### 3.4. Modulation of THz Waves in Hybrid Superconducting Photonic Circuits with Voltage

**Figure 6:**
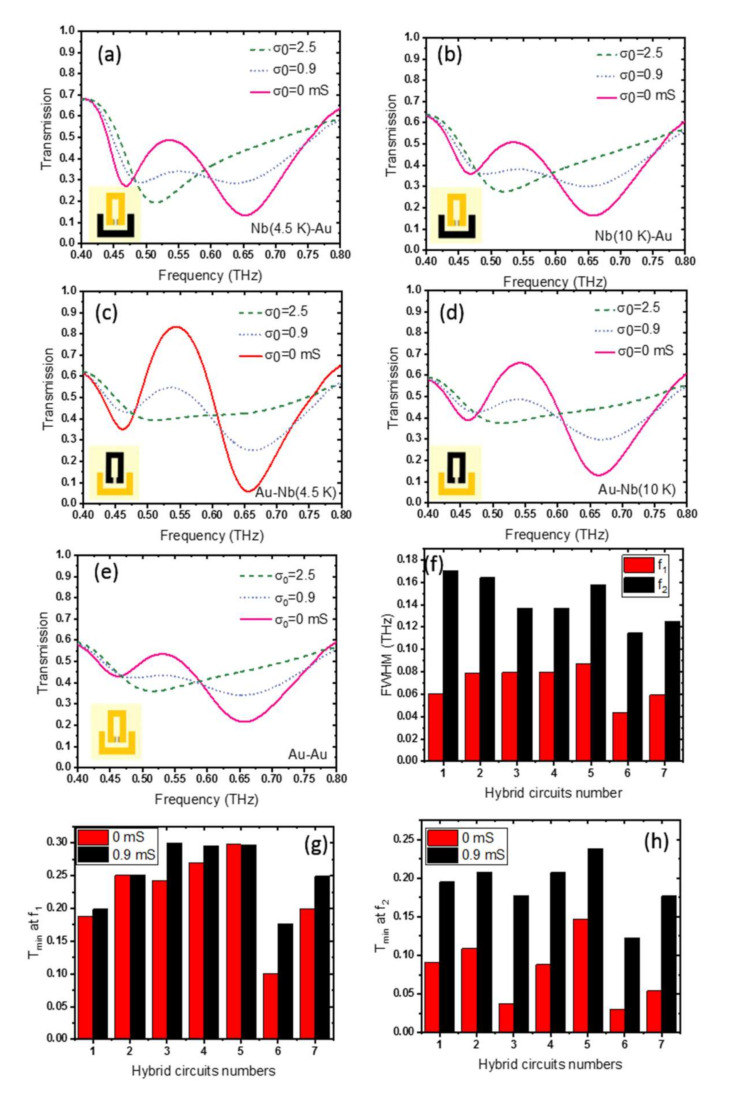
THz transmission of the hybrid photonic integrated circuit when the bright/dark resonators are set to be (**a**) Nb (at *T* = 4.5 K)/Au and (**b**) Nb (at *T* = 10 K)/Au at three different graphene conductivities. The same info is given in (**c**) and (**d**) but with Nb and Au replaced in the SRR unit-cells. Insets show one unit-cell, where Nb SRR is shown in black, and Au SRR is shown in yellow. (**e**) THz transmission of the photonic integrated circuit when the bright/dark resonators are both set to be Au. (**f**) FWHM at *σ*_0_ = 0 mS, (**g**) transmission minimum at the first resonance, and (**h**) transmission minimum at the second resonance of the hybrid circuit bright/dark resonators. Here, each device is labeled as 1-Nb (at *T* = 4.5 K)/Au, 2-Nb (at *T* = 10 K)/Au, 3-Au/Nb (at *T* = 4.5 K), 4-Au/Nb (at *T* = 10 K), 5-Au/Au, 6-Nb (at *T* = 4.5 K)/Nb (at *T* = 4.5 K), 7-Nb (at *T* = 10 K)/Nb (at *T* = 10 K) at *σ*_0_ = 0 mS and at *σ*_0_ = 0.9 mS.

We also compare the effect of the superconducting quantum phase on the THz response of such active devices with those of devices in the normal phase based on different materials such as gold [48]. Figure 6 summarizes the THz transmission of the hybrid photonic integrated circuit, when the bright/dark SRR resonators are set as: (a) Nb (at *T*= 4.5 K)/Au, (b) Nb (at *T* = 10 K)/Au, (c) Au/Nb (at *T* = 4.5 K), (d) Au/Nb (at *T* = 10 K), and (e) Au/Au, respectively. One can clearly find the role of superconductivity. Looking at Figure 6a–e, we find that the strongest response, in both resonance quality and EIT peak, is observed when both coupled resonators are made from Nb and when the Nb is set at *T* = 4.5 K < *T*c_Nb_. The strong difference between the THz responses of the Nb superconductor and Au resonators is originates from the difference in ohmic (nonradiative) losses of the resonators. The nonradiative losses depend on the resistance of the device material building blocks. The lower nonradiative losses of Nb at the superconducting state result in exciting sharper resonances [55,56].

The THz surface resistance of Nb is small, a value around *R*_s_ = 100 mΩ between *f* = 100 GHz and 600 GHz, at *T* = 5 K [13]. Replacing Nb in the bright resonator with Au will reduce the response (increase the FWHM), but this reduction is significant when Nb in the dark resonator is replaced by Au (see Figure 6f). The device transmission minimum at resonance frequencies will significantly weaken when the SRR structure is built from Au, as shown in Figure 6g,h, which retains large surface resistance at all temperatures.

### 3.5. The Equivalent RLC Circuit Model of Hybrid Graphene Superconducting THz Photonic Circuits

**Figure 7:**
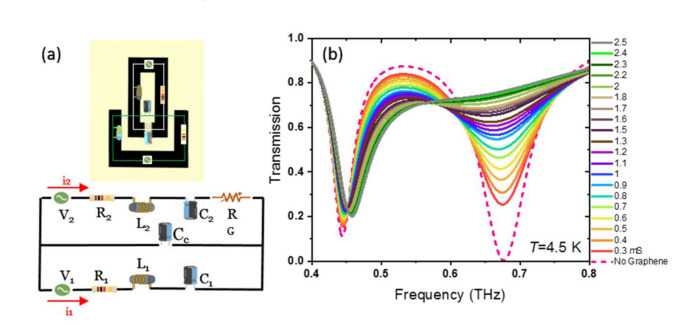
(**a**) The equivalent *RLC* circuit model of the hybrid graphene-Nb coupled resonators. (**b**) THz transmission and frequency tuning of the hybrid SRR for different graphene conductivities between *σ*_0_ = 0 and *σ*_0_ =2.5 mS obtained using the equivalent *RLC* model.

We use an equivalent lumped element circuit model to further investigate the resonance response and frequency tuning of the coupled resonators as a function of graphene conductivity. Figure 7a shows the *RLC* electrical circuit equivalent of the SRR. In the absence of coupling, each single SRR can be considered as an antenna with a frequency-dependent impedance. The electric circuit in Figure 7a, with the lower loop consisting of a resistance *R*_1_, an inductance *L*_1,_ and a capacitance *C*_1_, represents the bright resonator. The upper loop represents the dark resonator with *R*_2_, *L*_2,_ and *C*_2_ circuit elements. In the presence of coupling, due to the electric field of charges in close proximity between two bright and dark SRRs, a parallel coupling capacitor *C*_c_ can connect the two circuit loops. In the circuit, *R*_1_ and *R*_2_ illustrate the losses in each SRR; *L*_1_ and *L*_2_ represent the stored magnetic energy due to induced current in each SRR; *C*_1_ and *C*_2_ illustrate the energy stored in the finger of each bright and dark SRR due to accumulated charges. *R*_G_ accounts for the extra induced (resistive) losses as a result of the integration of graphene with Nb in the finger of the dark SRR. *V*_1_ and *V*_2_ in the circuit represent the electromotive force on the electrons due to the incident THz radiation in the bright and dark SRRs, respectively. We assume that the value of *V*_1_ is 0.65*V*_2_ as the bright SRR provides a larger coupling with the incident THz transmission. The inductance of each resonator *L*_i_ is defined as the ratio of magnetic field flow divided by current *J* density, as $L_{i}=\frac{\int H_{z}dxdy}{\int Jdxdy}$. The *z*-component of the magnetic field *H*_z_ and induced current density *J* for each SRR are obtained from the COMSOL simulation. After determining the inductance of each SRR, the capacitance is calculated from the equation $\omega_{r}=\frac{1}{\sqrt{L_{i}C}}$ [31,57].

A free fitting parameter as $C_{c}=20\;fF$ is used to obtain $C_{1}$ and $C_{2}$ from the equation $C=\frac{C_{i}+C_{c}}{C_{i}C_{c}}$. The resistance $R_{i}$ of each SRR is calculated from the equation $Q_{i}=\frac{1}{R_{i}}\sqrt{\frac{L_{i}}{C_{i}}}$ [58], where the quality factor of each ring is obtained from $Q_{i}=\frac{f_{r}}{\Delta f}$. Here, $f_{r}$ and Δf are resonance frequency and the FWHM of the resonance, respectively [31]. The obtained circuit element values for each loop are shown in Table 1. The dampening resistor *R*_G_ as a function of graphene conductivity *σ*_0_ is determined from the equation $Q=\frac{1}{R_{no}+R_{G}}\sqrt{\frac{L_{2}}{C_{2}}}$.

First, the quality factor of the resonance of a single dark SRR is determined by performing a COMSOL simulation. Then the resistance of the dark SRR without graphene (*R*_w_) is calculated. *R*_G_ changes from 2.5 Ω for *σ*_0_ = 0.3 mS to 25 Ω for *σ*_0_ = 2.5 mS. The current induced in the bright and dark SRRs (named as *i*_1_ and *i*_2_, respectively) will flow through the bottom and top loops. The voltages are related to currents through Kirchhoff’s law (see Appendix A Equation (A1)). Finally, transmission *T* can be obtained from $T=1-\frac{1}{2}\left(Re\;\left(V_{1}i_{1}^{*}+V_{2}i_{2}^{*}\right)\right)$, where the complex conjugates of the current induced in the bright and dark resonators are described by $i_{1}^{*}$ and $i_{2}^{*}$, respectively [46,59].

The modeled THz transmission of the hybrid graphene-Nb SRR as a function of frequency for different graphene DC conductivities (and also for when there is no graphene in the finger of the dark SRR) at *T* = 4.5 K is shown in Figure 7b. The electric field *E_gap_* across the capacitive gap of the Nb SRR and the graphene conductivity will determine the power dissipation and, therefore, the amount of resonance dampening. As *E_gap_* increases, *R*_G_ increases, and the second resonance quality decreases. One can see that the results of the THz transmission obtained from the *RLC* equivalent circuit model based on the destructive interference between *i*_1_ and *i*_2_ are in reasonably good agreement with the COMSOL simulation (see Figure 5a). Next, we focus on the EIT peak, which is observed between two bonding *f* ^−^ and anti-bonding *f* ^+^ resonances (see Figure 2). The EIT offers an extreme modification of the dispersion, resulting in the slow light effect. This slow light effect in hybrid metamaterial integrated circuits can be observed as a result of the group velocity alteration in the device.

### 3.6. Active Control of Slow Light Effect in Hybrid Superconducting THz Photonic Circuits

**Figure 8:**
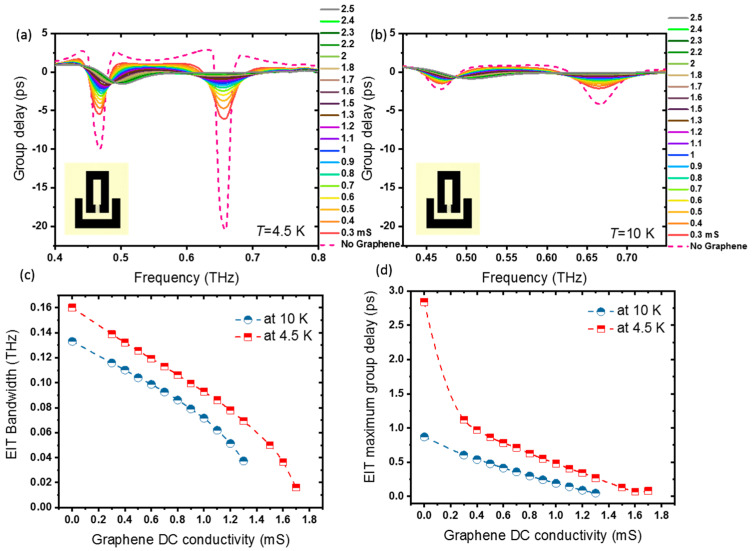
Group delay through the hybrid photonic integrated circuit for different graphene conductivities: at (**a**) *T* = 4.5 K, far from the Nb superconducting transition temperature *T*_c_, and (**b**) at *T* = 10 K, where Nb is no longer in the superconducting quantum phase. (**c**) The bandwidth of the transparency window and (**d**) EIT maximum group delay through the metamaterial photonic integrated circuit, with hybrid graphene-Nb SRRs, as a function of graphene conductivity at *T* = 4.5 K < *T*_c_ and at *T*= 10 K > *T*_c_.

Here, we demonstrate how to actively control slow light in hybrid graphene–superconductor resonators integrated with superconducting circuits. The group delay $t_{g}$, the time delay of a THz wave packet traveling through the device in comparison to air, is determined by $t_{g}=\frac{d\phi }{d\omega }$, where ϕ is the phase of the THz transmission, and *ω* is the angular frequency. Group delay is calculated relative to the bare substrate reference. Figure 8 shows the group delay through the device as a function of graphene conductivity for temperatures (a) *T* = 4.5 K < *T_c_* and (b) *T* = 10 K > *T_c_*. Maximum negative group delay dips are observed at around 0.46 THz and 0.66 THz at both temperatures; however, the group delay value and shape observed at *T* = 4.5 K < *T*_c_, where Nb is in the superconducting quantum phase, is much larger and sharper. The positive group delay between these frequencies indicates the slow light effect through the hybrid device. It can be seen that the delay time is quite loss-sensitive and is much larger when Nb is in the superconducting quantum phase at *T* = 4.5 K. A maximum positive group delay of 2.86 ps at 0.627 THz is observed when there is no graphene present in the Nb gap in the dark resonators (with Nb in its smallest THz surface resistance value). The maximum group delay is in the order of other superconducting metamaterials [36,60]. In comparison to the non-superconducting metamaterials with graphene, the group delay is larger or equal depending on the geometry [42,61,62]. On the other hand, in comparison with the same structure based on gold SRRs, our device shows a larger group delay [46]. The maximum positive slow light and the group delay values decrease as the conductivity of graphene increases (damping increases) for both temperatures. Figure 8c,d summarizes the bandwidth of the transparency window and EIT maximum group delay through the metamaterial photonic integrated circuit as a function of graphene conductivity at *T* = 4.5 K and *T* = 10 K. Our results indicate that the proposed hybrid photonic integrated circuit based on graphene-Nb SRR can be used as an efficient and tunable THz slow light modulator device.

**Figure 9:**
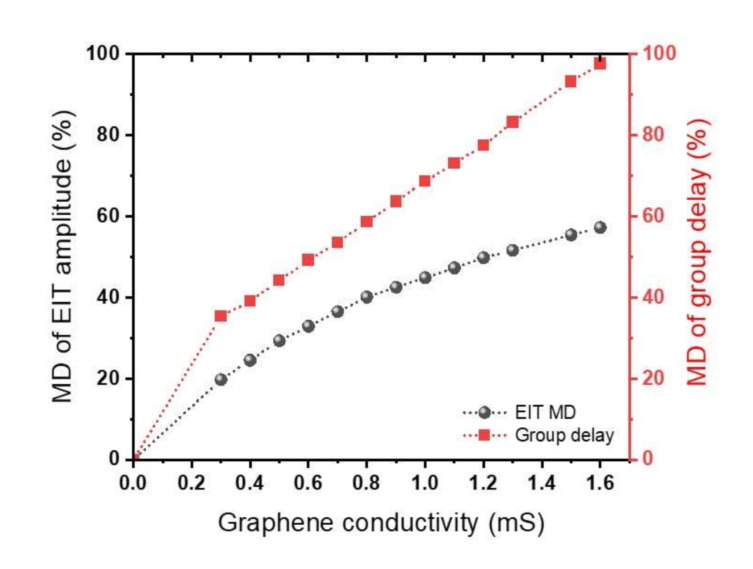
Modulation depth (MD) of the EIT and group delay as a function of graphene conductivity at *f* = 0.541 THz and *T* = 4.5 K.

Applying a voltage on the device pads can significantly change the amplitude of the EIT peak, rather than the resonance frequencies, as is clear from Figure 8a,b. To quantitatively investigate this variation, we introduced the modulation depth (MD) of the EIT amplitude as a function of graphene conductivity as $MD_{amplitude}=\left|\frac{T_{\left(0\;mS\right)}-T}{T_{\left(0\;mS\right)}}\right|$, where $T_{\left(0\;mS\right)}$ is transmission at σ0 = 0 mS, and T is transmission at the desired graphene conductivity. Both $T_{\left(0\;mS\right)}$ and T are obtained at the same frequency of f = 0.541 THz. The MD of the EIT amplitude (shown in Figure 9) increases with the graphene conductivity. It has a maximum value of 57.3% at *σ*_0_ = 1.6 mS for T = 4.5 K. As the graphene conductivity increases, the EIT transmission has an obvious decline that results in the MD growing. When *σ*_0_ becomes larger than 1.6 mS at *T* = 4.5 K, the EIT peak shows no more positive group delay. Positive group delay corresponds to the slowness of slow light devices. In addition, active modulation of slow light can be realized by graphene conductivity. The modulation depth of group delay is determined by $MD_{group\;delay}=\left|\frac{t_{g}{}_{\left(0\;mS\right)}-t_{g}}{t_{g}{}_{\left(0\;mS\right)}}\right|$. The MD of slow light shown in red displays an increase with graphene conductivity. The MD of group delay is as high as 97.61 % at *σ*_0_ = 1.6 mS for T = 4.5 K. 

**Figure 10:**
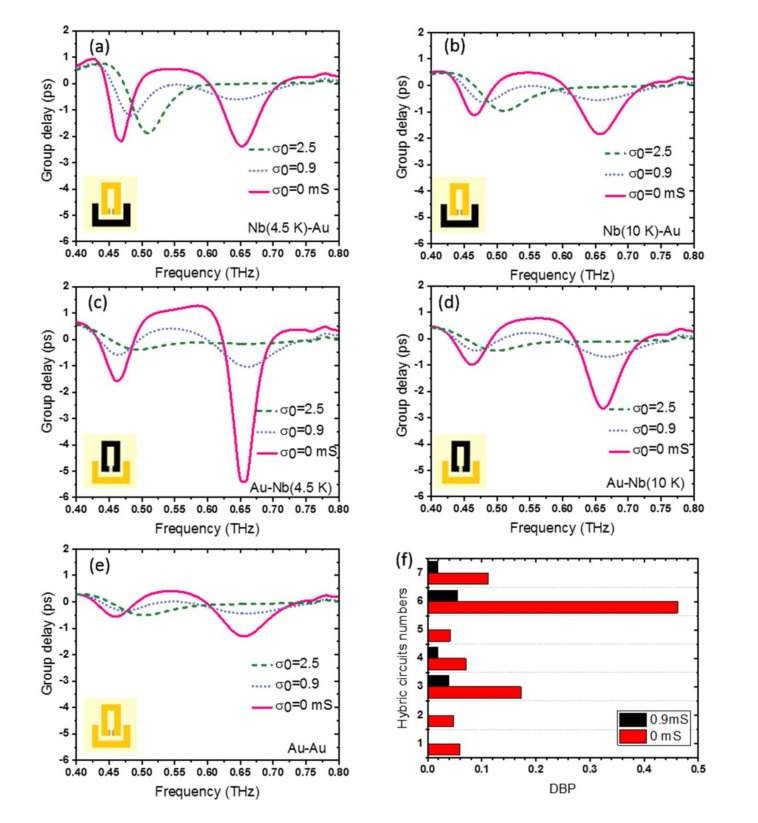
Group delay through the hybrid photonic integrated circuit for different graphene conductivity when the bright/dark resonators are set to be (**a**) Nb (at *T* = 4.5K)/Au and (**b**) Nb (at 10K)/Au. The same information is given in (**c**,**d**) but with Nb and Au replaced in the SRR unit-cells. Insets show one unit-cell, where Nb SRR is shown in black, and Au SRR is shown in yellow. (**e**) Group delay through the hybrid photonic integrated circuit for different graphene conductivity when the bright/dark resonators are both set as Au. (**f**) delay-bandwidth product of hybrid device bright/dark resonators for device # 1-Nb (at *T* = 4.5 K)/Au, 2-Nb (at *T* = 10 K)/Au, 3-Au/Nb (at *T* = 4.5 K), 4-Au/Nb (at *T* = 10 K), 5-Au/Au, 6-Nb (at *T* = 4.5 K)/Nb (at *T* = 4.5 K), 7-Nb (at *T* = 10 K)/Nb (at *T* = 10 K), at *σ*_0_ = 0 mS and *σ*_0_ = 0.9 mS.

Figure 10, with coupled SRR of different materials, provides further insight into the effect of the superconducting quantum phase on the group delay and slow light effect in such active devices. Here, the bright/dark SRRs are set as: (a) Nb (at *T* = 4.5 K)/Au, (b) Nb (at *T* = 10 K)/Au, (c) Au/Nb (at *T* = 4.5 K), (d) Au/Nb (at *T* = 10 K), and (e) Au/Au. One can clearly find the role of the superconducting quantum phase state. The strongest EIT response and slow light effect are observed when both coupled resonators are based on Nb and when the Nb is set at *T* = 4.5 K < Tc_Nb_. This is because at this temperature, the THz surface resistance of Nb is much smaller. Replacing Nb in the bright resonator with Au will reduce the group delay, but this reduction is considerable when Nb in the dark resonator is replaced by Au. The device response will significantly weaken when the SRR structure is built from Au, which retains large surface resistance at *T* = 4.5 K.

The maximum group delay, EIT window bandwidth, and maximum delay-bandwidth product (DBP) for different hybrid circuits, including device number 1-Nb (at *T* = 4.5 K)/Au, 2-Nb (at *T* = 10 K)/Au, 3-Au/Nb (at *T* = 4.5 K), 4-Au/Nb (at *T* = 10 K), 5-Au/Au, 6-Nb (at *T* = 4.5 K)/Nb (at *T* = 4.5 K), 7-Nb (at *T* = 10 K)/Nb (at *T* = 10 K), at σ_0_ =0 mS and σ_0_ = 0.9 mS, are calculated and presented in Table A1 and Table A2 of Appendix B. The DBPs for the different hybrid circuits are shown in Figure 10f. Device # 6 (Nb/Nb), at the lowest temperature of *T* = 4.5 K, has the largest DBP and is superior to the other superconducting metamaterials that has been reported [37]. Likewise, device # 5 (Au/Au) has the lowest DBP. Moreover, no EIT characteristic (0 DBP) is observed in the circuit when Nb is replaced by Au in the dark resonator.

## 4. Conclusions

We have proposed a voltage and temperature-controlled photonic integrated circuitry by the integration of graphene with an array of Nb subwavelength split-ring resonators in a superconducting circuit, and have reported the first demonstration of a hybrid graphene–superconductor THz metamaterial slow light device. Additionally, we have shown that an equivalent circuit model is a useful tool for active hybrid device optimization. Furthermore, we have demonstrated electromagnetic induced transparency, subradiant and superradiant resonances in such a novel class of hybrid photonic integrated circuits. The quantitative analysis has shown that the modulation depth of EIT transmission amplitude and slow light group delay can be realized via voltage and temperature. The maximum values of 57.3% and 97.61% have been obtained for the modulation depth of EIT and group delay with voltage. A comparison of Nb SRR-based devices with Au SRRs have shown the reduction of the maximum delay-bandwidth product from 0.46 to 0.04. Our devices, with their large and tunable slow light bandwidth, can pave the way for the realization of active optoelectronic modulators for applications in future quantum communication and computation systems.

## Figures and Tables

**Table 1:** The circuit parameters for lower and upper loops.

$R_{1}$ (Ω)	$L_{1}$ (pH)	$C_{1}$ (fF)	$R_{2}$ (Ω)	$L_{2}$ (pH)	$C_{2}$ (fF)
4.2712	0.2156	7.2453	6.9322	0.1276	5.9021

## Data Availability

The data presented in this paper can be accessed at https://www.repository.cam.ac.uk/handle/1810/330254 (accessed on 5 November 2021).

## Appendix A. Equivalent *RLC* Model

The current induced in the bright and dark SRRs (named as *i*_1_ and *i*_2_, respectively) that flows through the bottom and top loops can be obtained from the voltage in the bright SRR *V*_1_ and the dark SRR *V*_2_ based on Kirchhoff’s law:

(A1) $$\left[\begin{array}{c} i_{1}\\ i_{2} \end{array}\right]={\left[\begin{array}{cc} j\omega L_{1}+R_{1}+\frac{C_{1}+C_{c}}{j\omega C_{1}C_{c}} & \frac{1}{j\omega C_{c}}\\ \frac{1}{j\omega C_{c}} & j\omega L_{2}+R_{2}+R_{G}+\frac{C_{2}+C_{c}}{j\omega C_{2}C_{c}} \end{array}\right]}^{-1}\left[\begin{array}{c} V_{1}\\ V_{2} \end{array}\right]$$

## Appendix B. Group Delay Characteristics of Coupled SRR with Different Materials

**Table A1:** Maximum group delay, EIT bandwidth, and maximum group delay-EIT bandwidth product (DBP) of different hybrid circuits at *σ*_0_ = 0 mS.

@ 0 mS	Device Number	Max Group Delay (ps)	EIT Bandwidth (THz)	Max Delay-EIT BandWidth Product (DBP)
**Nb (4.5 K)/Au**	1	0.548	0.107	0.05863
**Nb (10 K)/Au**	2	0.444	0.106	0.04706
**Au/Nb (4.5 K)**	3	1.287	0.134	0.17245
**Au/Nb (10 K)**	4	0.565	0.125	0.07062
**Au/Au**	5	0.396	0.105	0.04158
**Nb (4.5 K)/Nb (4.5 K)**	6	2.87	0.161	0.46207
**Nb (10 K)/Nb (10 K)**	7	0.874	0.132	0.1118

**Table A2:** Maximum group delay, EIT bandwidth, and maximum group delay-EIT bandwidth product (DBP) of different hybrid circuits at *σ*_0_= 0.9 mS.

@ 0.9 mS	Device Number	Max Group Delay (ps)	EIT Bandwidth (THz)	Max Delay-EIT BandWidth Product (DBP)
**Nb (4.5 K)/Au ***	1	------	------	--------
**Nb (10 K)/Au ***	2	------	------	--------
**Au/Nb (4.5 K)**	3	0.410	0.094	0.03854
**Au/Nb (10 K)**	4	0.210	0.086	0.01806
**Au/Au**	5	0.114	0.016	0.00182
**Nb (4.5 K)/Nb (4.5 K)**	6	0.555	0.099	0.05494
**Nb (10 K)/Nb (10 K)**	7	0.242	0.079	0.01911

* These hybrid circuits show no positive group delay and, correspondingly, no EIT window at *σ*_0_ = 0.9 mS.
